# *cyclo*-Tetra­kis{μ-2,2′-dimethyl-1,1′-[2,2-bis­(bromo­meth­yl)propane-1,3-di­yl]di(1*H*-benzimidazole)-κ^2^ *N* ^3^:*N* ^3′^}tetra­kis­[bromidocopper(I)]

**DOI:** 10.1107/S160053681200102X

**Published:** 2012-01-14

**Authors:** Xing Wang, Chun-Bo Liu, Yong-Sheng Yan, Shen-Tang Wang, Ling Liu

**Affiliations:** a School of Chemistry and Chemical Engineering, Jiangsu University, Zhenjiang 212013, People’s Republic of China

## Abstract

The title compound, [Cu_4_Br_4_(C_21_H_22_Br_2_N_4_)_4_], features a macrocyclic Cu_4_ *L*_4_ ring system in which each Cu^I^ atom is coordinated by one bromide ion and two N atoms from two 2,2′-dimethyl-1,1′-[2,2-bis­(bromo­meth­yl)propane-1,3-di­yl]di(1*H*-benzimidazole) (*L*) ligands in a distorted trigonal–planar geometry. The *L* ligands adopt either a *cis* or *trans* configuration. The asymmetric unit contains one half-mol­ecule with the center of the macrocycle located on a crystallographic center of inversion. Each bromide ion binds to a Cu^I^ atom in a terminal mode and is oriented outside the ring. The macrocycles are inter­connected into a two-dimensional network by π–π inter­actions between benzimid­azole groups from different rings [centroid–centroid distance = 3.803 (5) Å.

## Related literature

For the synthesis of the organic ligand, see: Bai *et al.* (2010 ▶). For related structures, see: Zhu *et al.* (2005 ▶); Qi *et al.* (2008 ▶); Li & Du (2006 ▶); Peng *et al.* (2010 ▶).
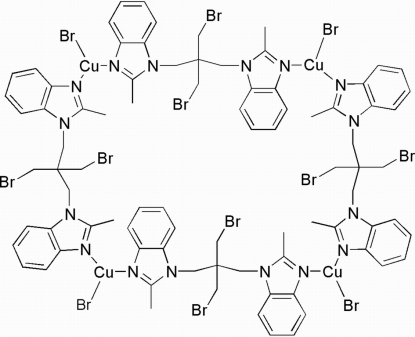


## Experimental

### 

#### Crystal data

[Cu_4_Br_4_(C_21_H_22_Br_2_N_4_)_4_]
*M* *_r_* = 2534.78
Triclinic, 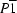

*a* = 11.585 (2) Å
*b* = 12.597 (3) Å
*c* = 15.273 (3) Å
α = 77.75 (3)°
β = 84.88 (3)°
γ = 89.54 (3)°
*V* = 2169.4 (8) Å^3^
*Z* = 1
Mo *K*α radiation
μ = 6.55 mm^−1^
*T* = 293 K
0.12 × 0.11 × 0.10 mm

#### Data collection

Rigaku Mercury CCD area-detector diffractometer
Absorption correction: multi-scan (*CrystalClear*; Rigaku, 2007 ▶) *T*_min_ = 0.458, *T*_max_ = 0.535
15281 measured reflections
7799 independent reflections
5648 reflections with *I* > 2σ(*I*)
*R*_int_ = 0.041

#### Refinement

*R*[*F* ^2^ > 2σ(*F* ^2^)] = 0.079
*wR*(*F* ^2^) = 0.177
*S* = 1.12
7799 reflections
523 parameters
H-atom parameters constrained
Δρ_max_ = 1.60 e Å^−3^
Δρ_min_ = −3.27 e Å^−3^

### 

Data collection: *CrystalClear* (Rigaku, 2007 ▶); cell refinement: *CrystalClear*; data reduction: *CrystalClear*; program(s) used to solve structure: *SHELXS97* (Sheldrick, 2008 ▶); program(s) used to refine structure: *SHELXL97* (Sheldrick, 2008 ▶); molecular graphics: *CrystalClear* (Rigaku, 2007 ▶) and *DIAMOND* (Brandenburg, 1998 ▶); software used to prepare material for publication: *SHELXTL* (Sheldrick, 2008 ▶).

## Supplementary Material

Crystal structure: contains datablock(s) global, I. DOI: 10.1107/S160053681200102X/im2344sup1.cif

Structure factors: contains datablock(s) I. DOI: 10.1107/S160053681200102X/im2344Isup2.hkl

Additional supplementary materials:  crystallographic information; 3D view; checkCIF report

## Figures and Tables

**Fig. 1.**
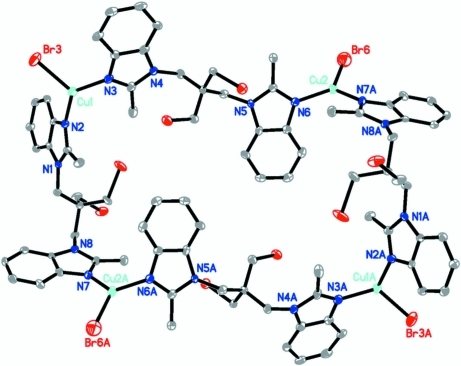
Crystal structure of the title compound (30% probability ellipsoids). All hydrogen atoms are omited for clarity.

**Fig. 2.**
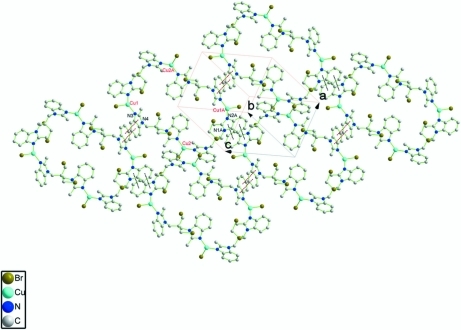
Two-dimensional layered structure constructed from different eight-membered Cu4L4 ring by π-π stacking interactions (dashed lines). All hydrogen atoms were omitted for clarity.

## supplementary crystallographic information

### Comment

Due to the charming structure topologies and applications in various areas, metal-organic frameworks have been widely developed (Zhu *et al.* 2005). A successful stategy for the construction of metal-organic frameworks is related to the coordination sites of linker and metal ion geometry. The flexible bis(imidazole) ligands are good candidates for constructing metal-organic networks, because of their numerous possible conformations, mainly *cis*- and *trans*-geometry (Qi *et al.* 2008). Copper (I) shows a variety of different coordination numbers such as two, three and four, and the interconversion between copper (I) and copper (II) makes their crystal structures even more versatile (Li *et al.* 2006; Peng *et al.* 2010). Here, we have used the flexible bis(benzimidazole) ligand, 1,1'-(2,2-bis(bromomethyl)propane-1,3-diyl) bis(2-methyl-1H- benzimidazol (L), and copper (II) bromide to obtain the title compound in which copper (II) was reduced to copper (I) under hydrothermal conditions.

In the crystal structure of the title compound, each copper (I) atom is coordinated by one bromide ion, and two N atoms from two ligands L, resulting in a trigonal planar geometry. The Cu—N distances range from 1.932 (8) to 2.003 (8) Å, while the distances of Cu1—Br3 and Cu2—Br6 are 2.381 (2) and 2.316 (3) Å, respectively. Two Cu1 atoms and two Cu2 atoms are linked by eight N atoms from four organic ligands L in an alternative *cis*-/*trans*-configuration, resulting in a centrosymmetric Cu_4_L_4_ ring. Only one half of the molecule is observed in the asymmetric unit, and there is a crystallographic center of inversion in the center of the macrocyclic molecule. Each bromide ion connects a copper (I) atom in a monodentate mode, oriented outside the ring (Fig. 2). The pitches of Cu1—Cu2 and Cu1—Cu2A are 12.738 (4) and 9.939 (4) Å, respectively. The rings are further interconnected to a two-dimensional network by π–π interactions. Around the ring, benzimidazol groups connected to N3 and N4, and benzimidazol groups based on N1 and N2, are stacked with a distance of 3.803 (5) Å (red dashed) and 3.613 (4) Å (black dashed), respectively (Fig. 2).

### Experimental

The organic ligand (L) was synthesized according to a previously reported procedure (Bai *et al.* 2010). A mixture of CuBr_2_ (22.365 mg, 0.1 mmol), and L (49.023 mg, 0.1 mmol) was dissolved in 10 mL of water of pH = 6. The resulting mixture was then transferred to a 25 mL Teflon–lined stainless steel reactor, and heated to 438 K for three days. After the reactor was slowly cooled to the room temperature yellow block-shaped crystals were obtained with a yield of 53 %.

### Refinement

Anisotropical displacement parameters were applied for all non-hydrogen atoms. Hydrogen atoms were positioned geometrically and refined in a riding model with C—H distances of 0.96, 0.97 and 0.93 Å for methyl groups, methylene groups and benzene rings and with *U*_iso_(H)=1.5*U*_eq_(CH_3_), *U*_iso_(H)=1.2*U*_eq_(CH_2_), *U*_iso_(H)=1.2*U*_eq_(CH), respectively.

### Crystal data

**Table:**

[Cu_4_Br_4_(C_21_H_22_Br_2_N_4_)_4_]	*Z* = 1
*M**_r_* = 2534.78	*F*(000) = 1240
Triclinic, *P*1	*D*_x_ = 1.940 Mg m^−^^3^
Hall symbol: -P 1	Mo *K*α radiation, λ = 0.71073 Å
*a* = 11.585 (2) Å	Cell parameters from 8554 reflections
*b* = 12.597 (3) Å	θ = 3.3–29.0°
*c* = 15.273 (3) Å	µ = 6.55 mm^−^^1^
α = 77.75 (3)°	*T* = 293 K
β = 84.88 (3)°	Prism, yellow
γ = 89.54 (3)°	0.12 × 0.11 × 0.10 mm
*V* = 2169.4 (8) Å^3^	

### Data collection

**Table:**

Rigaku DIFFRACTOMETER NAME? CCD area-detector diffractometer	7799 independent reflections
Radiation source: fine-focus sealed tube	5648 reflections with *I* > 2σ(*I*)
graphite	*R*_int_ = 0.041
Detector resolution: 28.5714 pixels mm^-1^	θ_max_ = 25.4°, θ_min_ = 3.1°
ω scans	*h* = −13→13
Absorption correction: multi-scan (*CrystalClear*; Rigaku, 2007)	*k* = −15→12
*T*_min_ = 0.458, *T*_max_ = 0.535	*l* = −18→16
15281 measured reflections	

### Refinement

**Table:**

Refinement on *F*^2^	Primary atom site location: structure-invariant direct methods
Least-squares matrix: full	Secondary atom site location: difference Fourier map
*R*[*F*^2^ > 2σ(*F*^2^)] = 0.079	Hydrogen site location: inferred from neighbouring sites
*wR*(*F*^2^) = 0.177	H-atom parameters constrained
*S* = 1.12	*w* = 1/[σ^2^(*F*_o_^2^) + (0.0217*P*)^2^ + 38.1476*P*] where *P* = (*F*_o_^2^ + 2*F*_c_^2^)/3
7799 reflections	(Δ/σ)_max_ < 0.001
523 parameters	Δρ_max_ = 1.60 e Å^−^^3^
0 restraints	Δρ_min_ = −3.27 e Å^−^^3^

### Special details

**Table:**

Geometry. All esds (except the esd in the dihedral angle between two l.s. planes) are estimated using the full covariance matrix. The cell esds are taken into account individually in the estimation of esds in distances, angles and torsion angles; correlations between esds in cell parameters are only used when they are defined by crystal symmetry. An approximate (isotropic) treatment of cell esds is used for estimating esds involving l.s. planes.
Refinement. Refinement of F^2^ against ALL reflections. The weighted R-factor wR and goodness of fit S are based on F^2^, conventional R-factors R are based on F, with F set to zero for negative F^2^. The threshold expression of F^2^ > 2sigma(F^2^) is used only for calculating R-factors(gt) etc. and is not relevant to the choice of reflections for refinement. R-factors based on F^2^ are statistically about twice as large as those based on F, and R- factors based on ALL data will be even larger.

### Fractional atomic coordinates and isotropic or equivalent isotropic displacement parameters (Å^2^)

**Table:**

	*x*	*y*	*z*	*U*_iso_*/*U*_eq_	
C1	−0.0662 (9)	−0.2709 (9)	0.6373 (7)	0.037 (3)	
H1A	−0.1298	−0.3034	0.6788	0.055*	
H1B	−0.0957	−0.2277	0.5846	0.055*	
H1C	−0.0217	−0.2256	0.6653	0.055*	
C2	0.0092 (8)	−0.3581 (8)	0.6113 (6)	0.026 (2)	
C3	0.0703 (8)	−0.5202 (8)	0.5981 (6)	0.021 (2)	
C4	0.0897 (9)	−0.6300 (8)	0.5968 (6)	0.028 (2)	
H4	0.0385	−0.6842	0.6282	0.034*	
C5	0.1869 (9)	−0.6541 (8)	0.5474 (7)	0.029 (2)	
H5	0.2018	−0.7264	0.5461	0.035*	
C6	0.2646 (9)	−0.5744 (9)	0.4990 (7)	0.030 (2)	
H6	0.3304	−0.5944	0.4671	0.036*	
C7	0.2445 (8)	−0.4654 (8)	0.4981 (6)	0.027 (2)	
H7	0.2953	−0.4116	0.4654	0.032*	
C8	0.1468 (8)	−0.4398 (8)	0.5471 (6)	0.023 (2)	
C9	0.2330 (12)	−0.1854 (9)	0.6870 (8)	0.050 (3)	
H9A	0.2670	−0.1815	0.7414	0.075*	
H9B	0.2532	−0.2530	0.6704	0.075*	
H9C	0.1502	−0.1810	0.6967	0.075*	
C10	0.2767 (9)	−0.0950 (8)	0.6148 (7)	0.029 (2)	
C11	0.3050 (9)	0.0145 (8)	0.4853 (7)	0.028 (2)	
C12	0.3069 (9)	0.0668 (9)	0.3958 (7)	0.033 (3)	
H12	0.2644	0.0407	0.3558	0.040*	
C13	0.3748 (10)	0.1593 (10)	0.3688 (8)	0.041 (3)	
H13	0.3755	0.1975	0.3095	0.050*	
C14	0.4429 (10)	0.1984 (9)	0.4270 (8)	0.041 (3)	
H14	0.4898	0.2595	0.4052	0.049*	
C15	0.4407 (9)	0.1468 (9)	0.5161 (8)	0.036 (3)	
H15	0.4849	0.1721	0.5555	0.043*	
C16	0.3700 (9)	0.0556 (8)	0.5447 (7)	0.030 (2)	
C17	0.4021 (9)	−0.0112 (8)	0.7096 (7)	0.028 (2)	
H17A	0.4804	0.0171	0.6924	0.034*	
H17B	0.4084	−0.0853	0.7430	0.034*	
C18	0.3425 (8)	0.0559 (8)	0.7744 (6)	0.024 (2)	
C19	0.2133 (8)	0.0289 (8)	0.8004 (7)	0.028 (2)	
H19A	0.1885	0.0603	0.8516	0.033*	
H19B	0.2049	−0.0494	0.8198	0.033*	
C20	0.3630 (8)	0.1758 (8)	0.7329 (7)	0.026 (2)	
H20A	0.3347	0.1912	0.6737	0.031*	
H20B	0.4459	0.1900	0.7249	0.031*	
C21	0.4077 (9)	0.0133 (8)	0.8599 (7)	0.029 (2)	
H21A	0.3835	−0.0614	0.8844	0.034*	
H21B	0.4898	0.0127	0.8412	0.034*	
C22	0.5738 (9)	0.1851 (10)	0.8873 (7)	0.037 (3)	
H22A	0.5820	0.1460	0.8396	0.056*	
H22B	0.6392	0.1709	0.9223	0.056*	
H22C	0.5702	0.2615	0.8622	0.056*	
C23	0.4659 (9)	0.1491 (8)	0.9457 (6)	0.026 (2)	
C24	0.3034 (9)	0.0598 (8)	1.0015 (6)	0.028 (2)	
C25	0.3275 (9)	0.1347 (9)	1.0528 (7)	0.031 (2)	
C26	0.2557 (10)	0.1443 (10)	1.1279 (7)	0.040 (3)	
H26	0.2710	0.1944	1.1622	0.048*	
C27	0.2074 (9)	−0.0117 (9)	1.0241 (7)	0.035 (3)	
H27	0.1926	−0.0633	0.9910	0.042*	
C28	0.1367 (10)	0.0006 (11)	1.0991 (8)	0.048 (3)	
H28	0.0710	−0.0434	1.1162	0.058*	
C29	0.1594 (11)	0.0753 (11)	1.1498 (8)	0.051 (3)	
H29	0.1091	0.0797	1.1998	0.061*	
C30	−0.2859 (10)	−0.4545 (9)	0.9838 (7)	0.035 (3)	
H30A	−0.3277	−0.4050	1.0146	0.053*	
H30B	−0.2084	−0.4274	0.9652	0.053*	
H30C	−0.2832	−0.5244	1.0236	0.053*	
C31	−0.3457 (9)	−0.4648 (8)	0.9028 (6)	0.027 (2)	
C32	−0.4755 (9)	−0.4457 (8)	0.8064 (6)	0.028 (2)	
C33	−0.3936 (8)	−0.5168 (8)	0.7814 (6)	0.023 (2)	
C34	−0.5741 (9)	−0.4194 (9)	0.7605 (7)	0.030 (2)	
H34	−0.6290	−0.3717	0.7777	0.036*	
C35	−0.4076 (9)	−0.5659 (8)	0.7100 (6)	0.027 (2)	
H35	−0.3539	−0.6152	0.6939	0.033*	
C36	−0.5865 (9)	−0.4672 (9)	0.6885 (7)	0.033 (2)	
H36	−0.6510	−0.4510	0.6562	0.039*	
C37	−0.5053 (9)	−0.5388 (9)	0.6633 (7)	0.031 (2)	
H37	−0.5162	−0.5693	0.6142	0.038*	
C38	−0.2155 (8)	−0.6045 (8)	0.8477 (6)	0.027 (2)	
H38A	−0.2383	−0.6648	0.8225	0.032*	
H38B	−0.2054	−0.6331	0.9107	0.032*	
C39	−0.0963 (8)	−0.5607 (8)	0.7996 (6)	0.026 (2)	
C40	−0.0547 (9)	−0.4732 (10)	0.8464 (8)	0.039 (3)	
H40A	−0.0692	−0.4991	0.9110	0.047*	
H40B	−0.1008	−0.4086	0.8297	0.047*	
C41	−0.1132 (8)	−0.5181 (9)	0.6990 (6)	0.028 (2)	
H41A	−0.1391	−0.5786	0.6754	0.033*	
H41B	−0.1755	−0.4659	0.6953	0.033*	
C42	−0.0157 (10)	−0.6584 (10)	0.8086 (7)	0.042 (3)	
H42A	0.0583	−0.6355	0.7753	0.050*	
H42B	−0.0486	−0.7130	0.7817	0.050*	
N1	−0.0138 (7)	−0.4666 (6)	0.6392 (5)	0.0233 (18)	
N2	0.1043 (7)	−0.3380 (7)	0.5564 (5)	0.0279 (19)	
N3	0.2474 (8)	−0.0801 (7)	0.5328 (6)	0.032 (2)	
N4	0.3491 (7)	−0.0150 (6)	0.6276 (5)	0.0265 (19)	
N5	0.3924 (7)	0.0722 (7)	0.9319 (5)	0.0263 (19)	
N6	0.4314 (7)	0.1893 (7)	1.0158 (5)	0.030 (2)	
N7	−0.4426 (7)	−0.4119 (7)	0.8828 (5)	0.0259 (19)	
N8	−0.3119 (7)	−0.5267 (6)	0.8428 (5)	0.0232 (18)	
Cu1	0.17897 (11)	−0.19933 (10)	0.48635 (8)	0.0321 (3)	
Cu2	0.51139 (12)	0.29469 (11)	1.06772 (9)	0.0328 (3)	
Br1	0.01050 (11)	−0.72422 (12)	0.93423 (8)	0.0536 (4)	
Br2	0.10711 (13)	−0.43322 (15)	0.81878 (9)	0.0755 (6)	
Br3	0.22461 (15)	−0.19296 (11)	0.33021 (9)	0.0632 (4)	
Br4	0.10892 (9)	0.07814 (9)	0.70714 (8)	0.0376 (3)	
Br5	0.29072 (10)	0.27524 (9)	0.80146 (8)	0.0367 (3)	
Br6	0.70608 (18)	0.25901 (17)	1.08162 (14)	0.0914 (6)	

### Atomic displacement parameters (Å^2^)

**Table:**

	*U*^11^	*U*^22^	*U*^33^	*U*^12^	*U*^13^	*U*^23^
C1	0.037 (6)	0.038 (7)	0.035 (6)	0.003 (5)	−0.001 (5)	−0.008 (5)
C2	0.027 (5)	0.028 (6)	0.022 (5)	0.000 (4)	−0.003 (4)	−0.003 (4)
C3	0.020 (5)	0.028 (5)	0.015 (5)	−0.001 (4)	−0.006 (4)	−0.003 (4)
C4	0.033 (6)	0.030 (6)	0.019 (5)	−0.006 (5)	−0.002 (4)	0.001 (4)
C5	0.037 (6)	0.021 (5)	0.027 (5)	0.004 (5)	−0.002 (5)	0.001 (4)
C6	0.028 (5)	0.039 (6)	0.023 (5)	0.005 (5)	−0.004 (4)	−0.008 (5)
C7	0.024 (5)	0.032 (6)	0.023 (5)	−0.002 (4)	−0.002 (4)	−0.004 (5)
C8	0.027 (5)	0.025 (5)	0.018 (5)	−0.001 (4)	−0.005 (4)	−0.007 (4)
C9	0.088 (10)	0.026 (6)	0.036 (7)	−0.015 (6)	−0.013 (7)	−0.003 (5)
C10	0.044 (6)	0.017 (5)	0.026 (5)	0.002 (5)	−0.003 (5)	−0.001 (4)
C11	0.030 (6)	0.025 (6)	0.027 (5)	0.006 (4)	0.000 (4)	−0.004 (5)
C12	0.037 (6)	0.031 (6)	0.031 (6)	0.007 (5)	0.006 (5)	−0.008 (5)
C13	0.049 (7)	0.036 (7)	0.034 (6)	0.007 (6)	0.009 (5)	0.001 (5)
C14	0.046 (7)	0.025 (6)	0.046 (7)	−0.003 (5)	0.019 (6)	−0.004 (5)
C15	0.037 (6)	0.030 (6)	0.043 (7)	−0.004 (5)	0.009 (5)	−0.019 (5)
C16	0.042 (6)	0.022 (5)	0.023 (5)	0.005 (5)	0.006 (5)	0.001 (4)
C17	0.030 (6)	0.023 (5)	0.035 (6)	0.001 (4)	−0.002 (5)	−0.013 (5)
C18	0.025 (5)	0.026 (5)	0.020 (5)	0.000 (4)	−0.004 (4)	−0.003 (4)
C19	0.028 (5)	0.024 (5)	0.028 (5)	−0.005 (4)	−0.002 (4)	0.001 (4)
C20	0.025 (5)	0.024 (5)	0.030 (5)	−0.003 (4)	−0.001 (4)	−0.008 (4)
C21	0.030 (6)	0.029 (6)	0.027 (5)	0.000 (5)	−0.001 (4)	−0.007 (5)
C22	0.033 (6)	0.044 (7)	0.035 (6)	−0.007 (5)	0.002 (5)	−0.010 (5)
C23	0.032 (6)	0.018 (5)	0.025 (5)	0.001 (4)	0.002 (4)	−0.003 (4)
C24	0.037 (6)	0.029 (6)	0.022 (5)	0.000 (5)	−0.008 (4)	−0.011 (4)
C25	0.031 (6)	0.030 (6)	0.031 (6)	−0.003 (5)	0.001 (5)	−0.005 (5)
C26	0.044 (7)	0.048 (7)	0.031 (6)	−0.005 (6)	−0.001 (5)	−0.016 (6)
C27	0.036 (6)	0.032 (6)	0.034 (6)	−0.011 (5)	0.002 (5)	−0.002 (5)
C28	0.039 (7)	0.055 (8)	0.045 (7)	−0.018 (6)	0.006 (6)	−0.004 (6)
C29	0.053 (8)	0.066 (9)	0.032 (6)	−0.008 (7)	0.013 (6)	−0.015 (6)
C30	0.043 (7)	0.036 (6)	0.028 (6)	0.002 (5)	−0.007 (5)	−0.008 (5)
C31	0.030 (6)	0.028 (6)	0.021 (5)	−0.006 (5)	−0.003 (4)	0.000 (4)
C32	0.034 (6)	0.023 (5)	0.024 (5)	−0.014 (5)	−0.003 (4)	−0.001 (4)
C33	0.015 (5)	0.026 (5)	0.023 (5)	−0.002 (4)	0.004 (4)	0.000 (4)
C34	0.023 (5)	0.031 (6)	0.036 (6)	−0.002 (4)	−0.005 (5)	−0.003 (5)
C35	0.027 (5)	0.029 (6)	0.026 (5)	−0.006 (4)	0.004 (4)	−0.008 (5)
C36	0.031 (6)	0.035 (6)	0.031 (6)	−0.013 (5)	−0.008 (5)	0.001 (5)
C37	0.031 (6)	0.043 (7)	0.022 (5)	−0.013 (5)	−0.001 (4)	−0.013 (5)
C38	0.027 (5)	0.033 (6)	0.019 (5)	−0.001 (5)	0.000 (4)	−0.003 (4)
C39	0.021 (5)	0.034 (6)	0.019 (5)	−0.004 (4)	−0.002 (4)	0.002 (4)
C40	0.033 (6)	0.049 (7)	0.035 (6)	−0.018 (5)	−0.004 (5)	−0.006 (6)
C41	0.017 (5)	0.038 (6)	0.027 (5)	−0.005 (4)	−0.001 (4)	−0.004 (5)
C42	0.031 (6)	0.059 (8)	0.028 (6)	0.003 (6)	−0.001 (5)	0.006 (6)
N1	0.029 (4)	0.024 (4)	0.016 (4)	−0.007 (4)	−0.003 (3)	−0.001 (3)
N2	0.028 (5)	0.032 (5)	0.023 (4)	−0.005 (4)	−0.002 (4)	−0.003 (4)
N3	0.039 (5)	0.029 (5)	0.026 (5)	−0.005 (4)	0.002 (4)	−0.005 (4)
N4	0.034 (5)	0.019 (4)	0.029 (4)	0.000 (4)	−0.004 (4)	−0.009 (4)
N5	0.027 (4)	0.027 (5)	0.027 (4)	−0.005 (4)	0.001 (4)	−0.012 (4)
N6	0.034 (5)	0.028 (5)	0.027 (5)	−0.003 (4)	−0.001 (4)	−0.008 (4)
N7	0.026 (4)	0.029 (5)	0.024 (4)	−0.002 (4)	−0.002 (4)	−0.007 (4)
N8	0.024 (4)	0.025 (5)	0.021 (4)	−0.002 (4)	0.000 (3)	−0.005 (4)
Cu1	0.0396 (8)	0.0265 (7)	0.0297 (7)	−0.0038 (6)	−0.0016 (6)	−0.0055 (6)
Cu2	0.0426 (8)	0.0274 (7)	0.0297 (7)	−0.0036 (6)	−0.0031 (6)	−0.0088 (6)
Br1	0.0370 (7)	0.0776 (10)	0.0342 (6)	0.0100 (6)	−0.0023 (5)	0.0140 (6)
Br2	0.0549 (9)	0.1109 (14)	0.0479 (8)	−0.0538 (9)	−0.0222 (7)	0.0207 (8)
Br3	0.1107 (13)	0.0428 (8)	0.0343 (7)	−0.0003 (8)	0.0047 (7)	−0.0089 (6)
Br4	0.0331 (6)	0.0377 (7)	0.0400 (6)	−0.0039 (5)	−0.0069 (5)	−0.0017 (5)
Br5	0.0378 (6)	0.0299 (6)	0.0440 (7)	0.0057 (5)	−0.0030 (5)	−0.0118 (5)
Br6	0.1004 (14)	0.0840 (13)	0.0890 (13)	−0.0020 (11)	−0.0091 (11)	−0.0162 (11)

### Geometric parameters (Å, °)

**Table:**

C1—C2	1.496 (14)	C23—N6	1.309 (12)
C1—H1A	0.9600	C23—N5	1.356 (12)
C1—H1B	0.9600	C24—C25	1.393 (14)
C1—H1C	0.9600	C24—N5	1.398 (12)
C2—N2	1.316 (12)	C24—C27	1.409 (14)
C2—N1	1.364 (12)	C25—C26	1.381 (14)
C3—N1	1.363 (12)	C25—N6	1.403 (13)
C3—C8	1.405 (13)	C26—C29	1.391 (17)
C3—C4	1.404 (14)	C26—H26	0.9300
C4—C5	1.366 (14)	C27—C28	1.383 (15)
C4—H4	0.9300	C27—H27	0.9300
C5—C6	1.395 (14)	C28—C29	1.380 (17)
C5—H5	0.9300	C28—H28	0.9300
C6—C7	1.388 (14)	C29—H29	0.9300
C6—H6	0.9300	C30—C31	1.501 (14)
C7—C8	1.375 (13)	C30—H30A	0.9600
C7—H7	0.9300	C30—H30B	0.9600
C8—N2	1.400 (12)	C30—H30C	0.9600
C9—C10	1.465 (15)	C31—N7	1.326 (13)
C9—H9A	0.9600	C31—N8	1.355 (12)
C9—H9B	0.9600	C32—C33	1.384 (14)
C9—H9C	0.9600	C32—C34	1.394 (14)
C10—N3	1.301 (13)	C32—N7	1.408 (12)
C10—N4	1.372 (13)	C33—N8	1.378 (12)
C11—C12	1.384 (14)	C33—C35	1.385 (13)
C11—N3	1.397 (13)	C34—C36	1.380 (14)
C11—C16	1.409 (15)	C34—H34	0.9300
C12—C13	1.378 (16)	C35—C37	1.394 (14)
C12—H12	0.9300	C35—H35	0.9300
C13—C14	1.405 (17)	C36—C37	1.384 (15)
C13—H13	0.9300	C36—H36	0.9300
C14—C15	1.375 (16)	C37—H37	0.9300
C14—H14	0.9300	C38—N8	1.476 (12)
C15—C16	1.385 (14)	C38—C39	1.552 (13)
C15—H15	0.9300	C38—H38A	0.9700
C16—N4	1.387 (12)	C38—H38B	0.9700
C17—N4	1.453 (12)	C39—C42	1.529 (15)
C17—C18	1.547 (13)	C39—C40	1.538 (15)
C17—H17A	0.9700	C39—C41	1.546 (13)
C17—H17B	0.9700	C40—Br2	1.932 (10)
C18—C20	1.521 (13)	C40—H40A	0.9700
C18—C19	1.536 (13)	C40—H40B	0.9700
C18—C21	1.560 (13)	C41—N1	1.467 (12)
C19—Br4	1.948 (10)	C41—H41A	0.9700
C19—H19A	0.9700	C41—H41B	0.9700
C19—H19B	0.9700	C42—Br1	1.971 (11)
C20—Br5	1.936 (9)	C42—H42A	0.9700
C20—H20A	0.9700	C42—H42B	0.9700
C20—H20B	0.9700	N2—Cu1	2.003 (8)
C21—N5	1.449 (12)	N3—Cu1	1.990 (9)
C21—H21A	0.9700	N6—Cu2	1.962 (8)
C21—H21B	0.9700	N7—Cu2^i^	1.932 (8)
C22—C23	1.484 (14)	Cu1—Br3	2.3806 (18)
C22—H22A	0.9600	Cu2—N7^i^	1.932 (8)
C22—H22B	0.9600	Cu2—Br6	2.316 (3)
C22—H22C	0.9600		
			
C2—C1—H1A	109.5	C25—C26—C29	116.9 (11)
C2—C1—H1B	109.5	C25—C26—H26	121.6
H1A—C1—H1B	109.5	C29—C26—H26	121.6
C2—C1—H1C	109.5	C28—C27—C24	115.0 (10)
H1A—C1—H1C	109.5	C28—C27—H27	122.5
H1B—C1—H1C	109.5	C24—C27—H27	122.5
N2—C2—N1	112.3 (9)	C27—C28—C29	122.8 (11)
N2—C2—C1	123.3 (9)	C27—C28—H28	118.6
N1—C2—C1	124.4 (9)	C29—C28—H28	118.6
N1—C3—C8	106.1 (8)	C28—C29—C26	121.7 (11)
N1—C3—C4	133.9 (9)	C28—C29—H29	119.1
C8—C3—C4	120.0 (9)	C26—C29—H29	119.1
C5—C4—C3	117.3 (9)	C31—C30—H30A	109.5
C5—C4—H4	121.3	C31—C30—H30B	109.5
C3—C4—H4	121.3	H30A—C30—H30B	109.5
C4—C5—C6	122.5 (10)	C31—C30—H30C	109.5
C4—C5—H5	118.7	H30A—C30—H30C	109.5
C6—C5—H5	118.7	H30B—C30—H30C	109.5
C7—C6—C5	120.6 (10)	N7—C31—N8	111.9 (8)
C7—C6—H6	119.7	N7—C31—C30	121.6 (9)
C5—C6—H6	119.7	N8—C31—C30	126.5 (9)
C8—C7—C6	117.6 (9)	C33—C32—C34	121.8 (9)
C8—C7—H7	121.2	C33—C32—N7	109.0 (9)
C6—C7—H7	121.2	C34—C32—N7	129.2 (10)
C7—C8—N2	129.7 (9)	N8—C33—C32	105.9 (8)
C7—C8—C3	121.9 (9)	N8—C33—C35	133.1 (9)
N2—C8—C3	108.4 (8)	C32—C33—C35	120.9 (9)
C10—C9—H9A	109.5	C36—C34—C32	117.0 (10)
C10—C9—H9B	109.5	C36—C34—H34	121.5
H9A—C9—H9B	109.5	C32—C34—H34	121.5
C10—C9—H9C	109.5	C33—C35—C37	117.5 (9)
H9A—C9—H9C	109.5	C33—C35—H35	121.3
H9B—C9—H9C	109.5	C37—C35—H35	121.3
N3—C10—N4	113.1 (9)	C34—C36—C37	121.6 (10)
N3—C10—C9	123.4 (10)	C34—C36—H36	119.2
N4—C10—C9	123.4 (9)	C37—C36—H36	119.2
C12—C11—N3	130.9 (10)	C36—C37—C35	121.2 (9)
C12—C11—C16	120.5 (10)	C36—C37—H37	119.4
N3—C11—C16	108.6 (8)	C35—C37—H37	119.4
C13—C12—C11	116.8 (11)	N8—C38—C39	116.9 (8)
C13—C12—H12	121.6	N8—C38—H38A	108.1
C11—C12—H12	121.6	C39—C38—H38A	108.1
C12—C13—C14	122.8 (11)	N8—C38—H38B	108.1
C12—C13—H13	118.6	C39—C38—H38B	108.1
C14—C13—H13	118.6	H38A—C38—H38B	107.3
C15—C14—C13	120.4 (11)	C42—C39—C40	112.3 (9)
C15—C14—H14	119.8	C42—C39—C41	108.8 (8)
C13—C14—H14	119.8	C40—C39—C41	113.7 (9)
C14—C15—C16	117.3 (11)	C42—C39—C38	106.3 (8)
C14—C15—H15	121.3	C40—C39—C38	107.8 (8)
C16—C15—H15	121.3	C41—C39—C38	107.5 (8)
C15—C16—N4	132.1 (10)	C39—C40—Br2	115.0 (8)
C15—C16—C11	122.1 (9)	C39—C40—H40A	108.5
N4—C16—C11	105.8 (9)	Br2—C40—H40A	108.5
N4—C17—C18	118.5 (8)	C39—C40—H40B	108.5
N4—C17—H17A	107.7	Br2—C40—H40B	108.5
C18—C17—H17A	107.7	H40A—C40—H40B	107.5
N4—C17—H17B	107.7	N1—C41—C39	118.3 (8)
C18—C17—H17B	107.7	N1—C41—H41A	107.7
H17A—C17—H17B	107.1	C39—C41—H41A	107.7
C20—C18—C19	111.9 (8)	N1—C41—H41B	107.7
C20—C18—C17	108.4 (8)	C39—C41—H41B	107.7
C19—C18—C17	114.2 (8)	H41A—C41—H41B	107.1
C20—C18—C21	114.2 (8)	C39—C42—Br1	113.4 (7)
C19—C18—C21	107.3 (8)	C39—C42—H42A	108.9
C17—C18—C21	100.4 (8)	Br1—C42—H42A	108.9
C18—C19—Br4	116.5 (7)	C39—C42—H42B	108.9
C18—C19—H19A	108.2	Br1—C42—H42B	108.9
Br4—C19—H19A	108.2	H42A—C42—H42B	107.7
C18—C19—H19B	108.2	C3—N1—C2	107.5 (8)
Br4—C19—H19B	108.2	C3—N1—C41	125.4 (8)
H19A—C19—H19B	107.3	C2—N1—C41	127.1 (8)
C18—C20—Br5	115.3 (6)	C2—N2—C8	105.6 (8)
C18—C20—H20A	108.4	C2—N2—Cu1	132.3 (7)
Br5—C20—H20A	108.4	C8—N2—Cu1	121.9 (6)
C18—C20—H20B	108.4	C10—N3—C11	106.1 (9)
Br5—C20—H20B	108.4	C10—N3—Cu1	121.7 (7)
H20A—C20—H20B	107.5	C11—N3—Cu1	129.2 (7)
N5—C21—C18	117.5 (8)	C10—N4—C16	106.4 (8)
N5—C21—H21A	107.9	C10—N4—C17	126.6 (8)
C18—C21—H21A	107.9	C16—N4—C17	126.8 (9)
N5—C21—H21B	107.9	C23—N5—C24	106.6 (8)
C18—C21—H21B	107.9	C23—N5—C21	125.5 (8)
H21A—C21—H21B	107.2	C24—N5—C21	127.8 (8)
C23—C22—H22A	109.5	C23—N6—C25	106.0 (9)
C23—C22—H22B	109.5	C23—N6—Cu2	128.5 (7)
H22A—C22—H22B	109.5	C25—N6—Cu2	125.4 (7)
C23—C22—H22C	109.5	C31—N7—C32	105.3 (8)
H22A—C22—H22C	109.5	C31—N7—Cu2^i^	128.3 (7)
H22B—C22—H22C	109.5	C32—N7—Cu2^i^	125.6 (7)
N6—C23—N5	112.9 (9)	C31—N8—C33	107.8 (8)
N6—C23—C22	123.2 (9)	C31—N8—C38	127.3 (8)
N5—C23—C22	123.9 (9)	C33—N8—C38	124.3 (8)
C25—C24—N5	105.8 (9)	N3—Cu1—N2	128.3 (3)
C25—C24—C27	122.4 (10)	N3—Cu1—Br3	115.4 (2)
N5—C24—C27	131.8 (9)	N2—Cu1—Br3	115.1 (2)
C26—C25—C24	121.1 (10)	N7^i^—Cu2—N6	127.2 (4)
C26—C25—N6	130.2 (10)	N7^i^—Cu2—Br6	118.6 (3)
C24—C25—N6	108.6 (9)	N6—Cu2—Br6	113.9 (3)
			
N1—C3—C4—C5	−179.2 (10)	C39—C41—N1—C3	84.9 (12)
C8—C3—C4—C5	2.5 (14)	C39—C41—N1—C2	−98.2 (12)
C3—C4—C5—C6	−0.6 (15)	N1—C2—N2—C8	0.2 (11)
C4—C5—C6—C7	−1.1 (16)	C1—C2—N2—C8	−178.0 (9)
C5—C6—C7—C8	0.8 (15)	N1—C2—N2—Cu1	175.1 (6)
C6—C7—C8—N2	−178.4 (9)	C1—C2—N2—Cu1	−3.1 (15)
C6—C7—C8—C3	1.2 (14)	C7—C8—N2—C2	−179.3 (10)
N1—C3—C8—C7	178.4 (9)	C3—C8—N2—C2	1.0 (10)
C4—C3—C8—C7	−2.9 (14)	C7—C8—N2—Cu1	5.2 (14)
N1—C3—C8—N2	−1.9 (10)	C3—C8—N2—Cu1	−174.5 (6)
C4—C3—C8—N2	176.8 (8)	N4—C10—N3—C11	1.4 (12)
N3—C11—C12—C13	−179.0 (10)	C9—C10—N3—C11	179.0 (10)
C16—C11—C12—C13	0.0 (15)	N4—C10—N3—Cu1	163.7 (6)
C11—C12—C13—C14	2.3 (16)	C9—C10—N3—Cu1	−18.7 (15)
C12—C13—C14—C15	−2.6 (17)	C12—C11—N3—C10	178.7 (11)
C13—C14—C15—C16	0.5 (16)	C16—C11—N3—C10	−0.4 (11)
C14—C15—C16—N4	179.0 (10)	C12—C11—N3—Cu1	18.3 (16)
C14—C15—C16—C11	1.7 (15)	C16—C11—N3—Cu1	−160.8 (7)
C12—C11—C16—C15	−2.1 (15)	N3—C10—N4—C16	−1.9 (12)
N3—C11—C16—C15	177.2 (9)	C9—C10—N4—C16	−179.5 (10)
C12—C11—C16—N4	−179.9 (9)	N3—C10—N4—C17	−176.3 (9)
N3—C11—C16—N4	−0.7 (11)	C9—C10—N4—C17	6.1 (16)
N4—C17—C18—C20	−73.9 (11)	C15—C16—N4—C10	−176.1 (11)
N4—C17—C18—C19	51.7 (12)	C11—C16—N4—C10	1.4 (11)
N4—C17—C18—C21	166.1 (9)	C15—C16—N4—C17	−1.6 (17)
C20—C18—C19—Br4	51.5 (10)	C11—C16—N4—C17	175.9 (9)
C17—C18—C19—Br4	−72.1 (10)	C18—C17—N4—C10	−96.5 (12)
C21—C18—C19—Br4	177.6 (7)	C18—C17—N4—C16	90.1 (12)
C19—C18—C20—Br5	51.1 (10)	N6—C23—N5—C24	−1.7 (12)
C17—C18—C20—Br5	177.9 (6)	C22—C23—N5—C24	178.8 (10)
C21—C18—C20—Br5	−71.1 (9)	N6—C23—N5—C21	−179.8 (9)
C20—C18—C21—N5	52.5 (11)	C22—C23—N5—C21	0.6 (16)
C19—C18—C21—N5	−72.2 (11)	C25—C24—N5—C23	2.0 (11)
C17—C18—C21—N5	168.2 (8)	C27—C24—N5—C23	−176.3 (11)
N5—C24—C25—C26	179.9 (10)	C25—C24—N5—C21	−179.9 (9)
C27—C24—C25—C26	−1.6 (17)	C27—C24—N5—C21	1.8 (18)
N5—C24—C25—N6	−1.6 (12)	C18—C21—N5—C23	−97.2 (12)
C27—C24—C25—N6	176.8 (10)	C18—C21—N5—C24	85.0 (12)
C24—C25—C26—C29	0.4 (17)	N5—C23—N6—C25	0.6 (12)
N6—C25—C26—C29	−177.7 (12)	C22—C23—N6—C25	−179.8 (10)
C25—C24—C27—C28	2.1 (16)	N5—C23—N6—Cu2	175.8 (7)
N5—C24—C27—C28	−179.9 (11)	C22—C23—N6—Cu2	−4.6 (15)
C24—C27—C28—C29	−1.5 (18)	C26—C25—N6—C23	178.9 (12)
C27—C28—C29—C26	0(2)	C24—C25—N6—C23	0.7 (12)
C25—C26—C29—C28	0.2 (19)	C26—C25—N6—Cu2	3.5 (17)
C34—C32—C33—N8	−177.7 (9)	C24—C25—N6—Cu2	−174.7 (7)
N7—C32—C33—N8	0.5 (10)	N8—C31—N7—C32	2.4 (11)
C34—C32—C33—C35	−0.9 (15)	C30—C31—N7—C32	−177.1 (9)
N7—C32—C33—C35	177.3 (8)	N8—C31—N7—Cu2^i^	−167.7 (6)
C33—C32—C34—C36	−0.2 (15)	C30—C31—N7—Cu2^i^	12.8 (14)
N7—C32—C34—C36	−178.0 (10)	C33—C32—N7—C31	−1.8 (11)
N8—C33—C35—C37	177.5 (10)	C34—C32—N7—C31	176.3 (10)
C32—C33—C35—C37	1.7 (14)	C33—C32—N7—Cu2^i^	168.7 (6)
C32—C34—C36—C37	0.5 (15)	C34—C32—N7—Cu2^i^	−13.3 (15)
C34—C36—C37—C35	0.3 (16)	N7—C31—N8—C33	−2.2 (11)
C33—C35—C37—C36	−1.4 (15)	C30—C31—N8—C33	177.3 (10)
N8—C38—C39—C42	−175.7 (8)	N7—C31—N8—C38	−173.6 (8)
N8—C38—C39—C40	63.7 (11)	C30—C31—N8—C38	5.9 (16)
N8—C38—C39—C41	−59.3 (11)	C32—C33—N8—C31	1.0 (10)
C42—C39—C40—Br2	48.3 (11)	C35—C33—N8—C31	−175.3 (10)
C41—C39—C40—Br2	−75.8 (10)	C32—C33—N8—C38	172.7 (8)
C38—C39—C40—Br2	165.1 (7)	C35—C33—N8—C38	−3.6 (16)
C42—C39—C41—N1	−68.5 (12)	C39—C38—N8—C31	−93.0 (11)
C40—C39—C41—N1	57.5 (12)	C39—C38—N8—C33	97.0 (11)
C38—C39—C41—N1	176.8 (8)	C10—N3—Cu1—N2	18.6 (10)
C40—C39—C42—Br1	54.8 (10)	C11—N3—Cu1—N2	176.3 (8)
C41—C39—C42—Br1	−178.4 (7)	C10—N3—Cu1—Br3	−148.1 (7)
C38—C39—C42—Br1	−63.0 (10)	C11—N3—Cu1—Br3	9.7 (10)
C8—C3—N1—C2	2.0 (10)	C2—N2—Cu1—N3	65.2 (10)
C4—C3—N1—C2	−176.5 (10)	C8—N2—Cu1—N3	−120.6 (7)
C8—C3—N1—C41	179.5 (8)	C2—N2—Cu1—Br3	−128.0 (8)
C4—C3—N1—C41	1.0 (16)	C8—N2—Cu1—Br3	46.1 (8)
N2—C2—N1—C3	−1.5 (11)	C23—N6—Cu2—N7^i^	141.2 (8)
C1—C2—N1—C3	176.7 (9)	C25—N6—Cu2—N7^i^	−44.5 (10)
N2—C2—N1—C41	−178.9 (8)	C23—N6—Cu2—Br6	−45.3 (9)
C1—C2—N1—C41	−0.7 (15)	C25—N6—Cu2—Br6	129.1 (8)

Symmetry codes: (i) −*x*, −*y*, −*z*+2.

## References

[bb1] Bai, H.-Y., Xia, D.-C. & Ma, J.-F. (2010). *Z. Kristallogr. New Cryst. Struct.* **225**, 101–102.

[bb2] Brandenburg, K. (1998). *DIAMOND* Crystal Impact GbR, Bonn, Germany.

[bb3] Li, T. & Du, S.-W. (2006). *Acta Cryst.* E**62**, m1812–m1813.

[bb4] Peng, R., Li, M. & Li, D. (2010). *Coord. Chem. Rev.* **254**, 1–18.

[bb5] Qi, Y., Luo, F., Batten, S. R., Che, Y.-X. & Zheng, J.-M. (2008). *Cryst. Growth Des.* **8**, 2806–2813.

[bb6] Rigaku (2007). *CrystalClear* Rigaku/MSC Inc., The Woodlands, Texas, USA.

[bb7] Sheldrick, G. M. (2008). *Acta Cryst.* A**64**, 112–122. 10.1107/S0108767307043930 18156677

[bb8] Zhu, H.-F., Fan, J., Okamura, T.-A., Sun, W.-Y. & Ueyama, N. (2005). *Cryst. Growth Des.* **5**, 289–294.

